# Clinical Outcomes of Descemet Membrane Endothelial Keratoplasty During the Surgeon Learning Curve Versus Descemet Stripping Endothelial Keratoplasty Performed at the Same Time

**DOI:** 10.4172/2155-9570.1000599

**Published:** 2016-09-21

**Authors:** Jennifer Rose-Nussbaumer, Shashi Alloju, Winston Chamberlain

**Affiliations:** 1 Francis I. Proctor Foundation; San Francisco, CA, USA; 2 Department of Ophthalmology, University of California San Francisco, San Francisco, CA, USA; 3 Department of Ophthalmology, Oregon Health Sciences University, Portland, OR, USA

**Keywords:** Endothelial keratoplasty, Fuch’s dystrophy, Descemet membrane endothelial keratoplasty, Descemet stripping endothelial keratoplasty, Corneal transplantation

## Abstract

**Purpose:**

To compare outcomes after Descemet Membrane Endothelial Keratoplasty (DMEK) and traditional Descemet Stripping Endothelial Keratoplasty (DSEK) during the surgeon’s DMEK learning curve in a prospective, non-randomized, consecutive, interventional case series.

**Methods:**

Consecutive patients presenting to the university eye clinics and undergoing endothelial keratoplasty were included. Data including patient demographics, visual acuity, endothelial cell counts and complications were recorded at baseline, as well as 3 and 6 months post-operatively. The primary outcome for this study was BSCVA at 6 months. Pre-specified secondary outcomes included endothelial cell counts and complication rates.

**Results:**

A total of 60 eyes of 42 consecutive patients met inclusion criteria, underwent endothelial keratoplasty, and were included in this analysis. Of these, 18 eyes of 14 patients had DSEK while 42 eyes of 28 patients had DMEK. After controlling for baseline visual acuity, study participants undergoing DMEK had a statistically significant approximately half-line improvement in visual acuity compared with DSEK at 3 months (P=0.05) but not at 6 months (P=0.22). DMEK patients experienced an average of 43% endothelial cell loss compared with 25% in DSEK. There were 5 primary graft failures after DMEK compared with 0 after DSEK and but this was not a statistically significant difference (P=0.09).

**Conclusion:**

During the surgeon’s DMEK learning curve there was some evidence of improved visual acuity outcomes in DMEK. We observed worse 6-month endothelial cell loss among DMEK patients; however this may improve with surgeon experience.

## Introduction

Posterior lamellar keratoplasty techniques have evolved rapidly in recent years and Descemet Membrane Endothelial Keratoplasty (DMEK) has gained popularity [1]. Recent studies suggest that near anatomic replacement of endothelial tissue produces improved visual acuity results compared to Descemet Stripping Endothelial Keratoplasty (DSEK) [2]. However, according to the Eye Bank Association of America, DMEK still accounted for less than 15% of endothelial keratoplasties in the United States in 2015, while DSEK accounted for about 50% of all corneal transplants [3,4].

This suggests that the majority of endothelial keratoplasty (EK) surgeons in the United States have not yet adopted DMEK or are early on the DMEK learning curve). Experienced EK surgeons without fellowship training in DMEK may be reluctant to adopt the newer technique since they have excellent and reliable results with DSEK. The goal of this study is to provide both cornea specialists and patients with information on clinical outcomes they can expect during the DMEK learning curve compared with traditional DSEK. In this study we prospectively evaluate 6-month clinical outcomes of the first 42 consecutive DMEKs performed at one center versus 18 consecutive DSEK surgeries performed during the same time period on patients with Fuchs dystrophy and good visual potential.

## Methods

In this prospective, non-randomized, interventional series, consecutive patients presenting to Oregon Health Sciences University cornea clinics with Fuchs Endothelial Dystrophy (FED) who underwent endothelial keratoplasty (EK) with one surgeon (WC) were included. Exclusion criteria included patients with pre-existing conditions likely to affect visual acuity such as amblyopia, glaucoma, macular degeneration and macular edema or prior intraocular surgery other than cataract surgery.

Study participants were examined at enrollment, and post-operatively at 3 and 6 months. Data including patient demographics, visual acuity and refractive outcomes were collected. The primary outcome for this study was best spectacle-corrected visual acuity (BSCVA) at 6 months with intent to treat analysis. Therefore, we included actual 3 and 6-month visual acuity results even if they had primary graft failure requiring repeat endothelial keratoplasty. Pre-specified secondary outcomes included endothelial cell count at 6 months, as well as complications such as re-bubble rate, primary graft failure and graft rejection.

BSCVA was measured by Snellen chart. Baseline specular endothelial microscopy was performed by the eye bank (CellCheck EB-10, Konan Medical, Irvine, CA) and follow up counts were measured on clinical specular device SPS-2000P (Topcon, Oakland, NJ). Signed consent was obtained from all study participants. The study was approved by the institutional review board of the Oregon Health Sciences University and adhered to the Declaration of Helsinki. All surgeries were performed under the supervision of an experienced surgeon (W.C.).

## Patient selection

Study participants were not randomized to a treatment arm. Instead, they were given the option of DSEK or DMEK after a thorough discussion, including risks and benefits of each surgery, outcomes in the literature, and the surgeon’s early experience with the DMEK procedure. Since patients self-selected into their preferred treatment arm, this resulted in a disparity between arms.

### Surgery

All DSEK surgeries used standardized forceps insertion technique. All patients underwent previous or simultaneous non-complicated cataract surgery with phaco-emusification through a 2.75 mm limbal based 3-plane incision. An 8.0–8.5 mm area of host descemet membrane was stripped under Healon GV (AMO, Santa Ana, CA) using a reverse sinskey hook and pealed with a descemet stripper. The area of stripped descemet membrane was equal in diameter to the donor corneal graft. Healon GV was thoroughly evacuated from the eye with irrigation and aspiration and the diamond dusted I/A tip was used to gently score the peripheral stroma. Pre-cut corneal tissue, prepared by Portland Lions VisionGift eyebank, was trephined to 8.0–8.5 mm using a Barron-Hessburg punch (Katena Products, Denville, NJ). The endothelial disc was gently separated from the remaining donor cornea using the small end of a Paton spatula and folded into a 70/30 taco configuration. It was then grasped with Charlie 1 DSEK forceps (Storz (Bausch & Lomb, Bridgewater, NJ)) and inserted through a limbal-based 3 plain corneal incision that had been extended to 5.0 mm prior to insertion. The incision was closed with 10.0 nylon suture(s) and filtered air was injected to achieve a partial air fill. Gentle fluid waves and stroking motions on the surface of the cornea were used to unfold and center the graft. A full air fill was performed to a pressure of approximately 30–40 mmHg for 10 minutes and then the air bubble was reduced to 80% volume and the anterior chamber to physiologic pressure. One drop of 1% Atropine was used to dilate the pupil. The patient was asked to position horizontally laying face up for approximately 24 hours post-operatively.

For DMEK surgeries, all but one patient underwent previous or simultaneous non-complicated cataract surgery with phacoemulsification through a 2.75 clear corneal incision. Inferior iridotomies were made by passing a 30 g needle through the inferior peripheral iris in 3 separate locations. Other than the peripheral iridotomies, recipient preparation and stripping of descemet membrane was performed as described previously for DSEK. Pre-peeled corneal tissue, prepared by Portland Lions VisionGift eye bank, Portland Oregon as previously described [5], was stained with Vision Blue (DORC, Zuidland, Netherlands) to identify the edge of descemet membrane. The cornea was trephined to 7.75–8.25 mm using a Barron punch and the central descemet disc was separated from stroma under Optisol GS (SCUBA technique [6]) by gently lifting the graft from descemet side with a single tine of a McPherson tier and then grasping in one peripheral point to complete the peel. The scrolled central disc was stained in Vision blue (90% and 10% Optisol GS) for 5.0 min. Trypan was rinsed with balanced salt solution. For the first 20 cases, using standardized techniques, the grafts were picked up at the edge by McPherson tiers and front end loaded into a truncated AMO intraocular lens injector (Abbott Medical Optics, Santa Ana, CA) attached with polypropylene tubing to a 3.0 cc syringe that was preloaded with BSS. In the later 20 cases, the graft was gently aspirated into a modified Jones Tube in the standardized technique as described previously [7]. The clear corneal wound was extended to 3.2–3.5 mm for the Jones tube and the grafts were injected into the anterior chamber. The incision was closed with 10.0 nylon suture(s) and the chamber was shallowed. Fluid waves were used to orient the graft and gentle peripheral corneal taps were used to unfurl the graft and centralize it under the host. A full air fill was performed to a pressure of approximately 30–40 mmHg for 10 minutes and then the air bubble was reduced to 80% volume and the anterior chamber to physiologic pressure. One drop of 1% Atropine was used to dilate the pupil. The patient was asked to position horizontally laying face up for approximately 24 hours post-operatively. Initially every small detachment was re-bubbled, however after 20 DMEK cases, grafts were re-bubbled only if a greater than 20% detachment or detachment involving the visual axis was noted.

### Statistical analysis

The Mann-Whitney U test was performed to analyze differences in baseline characteristics between groups. We assessed the association between BSCVA and type of endothelial keratoplasty using multiple linear regressions and controlling for baseline visual acuity. We used intent to treat analysis; therefore, if there was primary graft failure that required repeat endothelial keratoplasty, the actual 3 and 6 month visual acuities after initial keratoplasty were used for analysis. As a sensitivity analysis we used a generalized linear mixed model to allow for non-independence of visual acuity measurements over time and within patient. Similarly we performed multiple linear regression to analyze the association between type of keratoplasty and endothelial cell loss with a term for baseline endothelial cell count. Mann-Whitney U test was performed to analyze differences in complication rates, such a re-bubble and graft failure. Statistical significance was defined by an alpha of <0.05 after Holm-Šidák adjustment for multiple comparisons. All analyses were conducted using Stata version 13.0.

## Results

A total of 234 endothelial keratoplasties were performed between May 2009 and May 2014. Of these 60 eyes of 42 consecutive patients with FED met inclusion criteria, and were included in this analysis. Of these, 18 eyes of 14 patients (7 men and 7 women) underwent DSEK. They had a mean age of 68 (SD 6.5) and mean baseline visual acuity was 0.35 logMAR (SD 0.25). Median baseline pachymetry was 616 μm (SD 40). DSEK was combined with phaco-emulsification in 16/18 cases (89%), and one iris-sutured intraocular lens (5.6%). The DSEK grafts were pre-cut by the eye bank with a mean graft thickness of 135 μm (SD 25; Range 106 to 186). DSEK graft thickness did not predict visual acuity at 6 months in our analysis (Coef. 0.00; 95% CI −0.002 to 0.001) and was therefore not included in the final statistical model. Mean pre-operative endothelial cell counts, as measured by the Eye Bank were 2757 cells/mm^2^ (SD 244; Range 2635–2879). Complications included one graft re-bubble (5.6%).

Forty-two eyes of 28 patients (8 men and 20 women) underwent DMEK. They had a mean age of 69 (SD 9.8) and mean baseline visual acuity of 0.38 (SD 0.22). Mean baseline pachymetry was 641 (SD 63) and mean baseline endothelial cell count was 2773 cells/mm^2^ (SD 224). These patients had phaco-emulsification at the time of endothelial keratoplasty in 21 cases (50%). Table 1 compares baseline characteristics of study participants. The only statistically significant difference between groups was the rate of concurrent phacoemulsification after Holms-Šidák comparison for multiple comparisons.

Table 2 outlines visual acuity outcomes and endothelial cell loss between the two groups. Baseline visual acuity was a statistically significant predictor of visual acuity at 3 months (Coef 0.26, 95% CI 0.10 to 0.40; P<0.001) and 6 months (Coef 0.19, 95% CI 0.04 to 0.34; P=0.002). Mean 3-month visual acuity among study participants receiving DSEK was LogMAR 0.18 (SD 0.07) and DMEK 0.13 (SD 0.14). After controlling for baseline visual acuity, study participants undergoing DMEK had 0.06 LogMAR lines of visual acuity improvement at 3-months compared with DSEK and this difference was statistically significant (95% CI −0.13 to 0.004; P=0.05).

**Figure 1:**
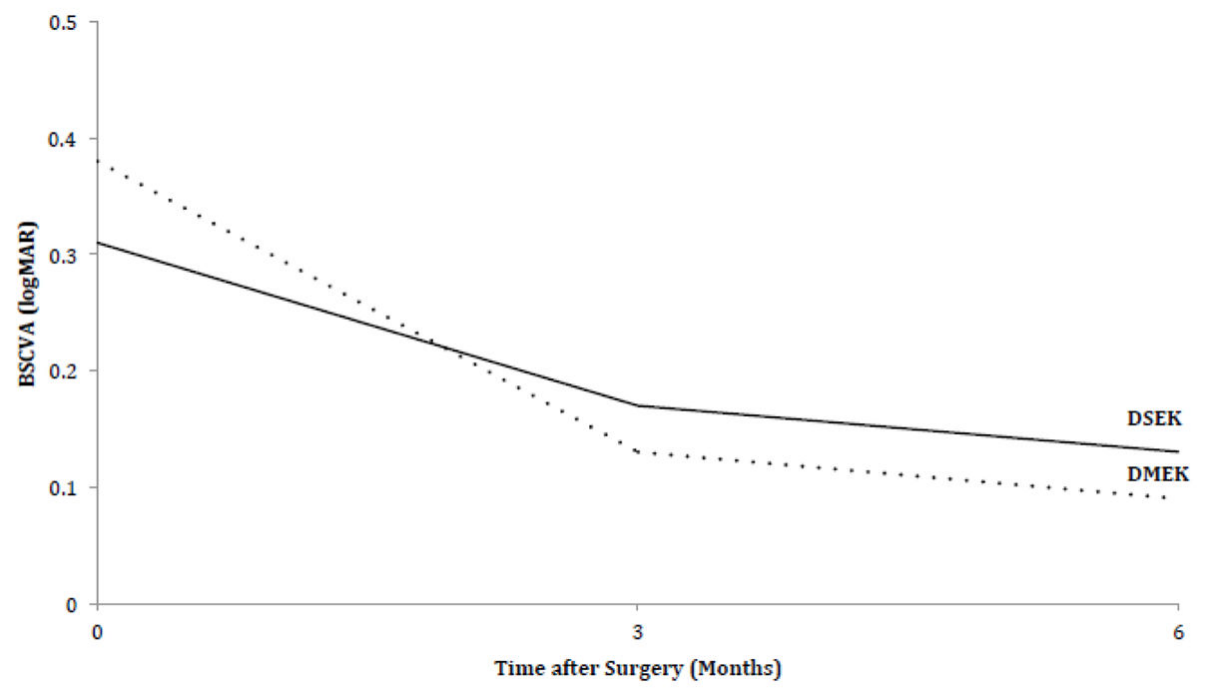
Visual Acuity. 6-month visual acuity improvement from baseline on logarithm of the minimum angle of resolution (logMAR) scale in study participants undergoing endothelial keratoplasty. BSCVA: Best Spectacle-Corrected Visual Acuity; DMEK: Descemet Membrane Endothelial Keratoplasty; DSEK: Descemet Stripping Endothelial Keratoplasty.

Mean 6-month visual acuity among study participants receiving DSEK was LogMAR 0.14 (SD 0.11) and DMEK was 0.09 (SD 0.12). After controlling for baseline visual acuity study participants receiving DMEK had 0.04 logMAR lines of visual acuity improvement compared with DSEK but this was no longer a statistically significant difference (95% CI −0.10 to 0.02; P=0.22). Figure 1 compares 6-month visual acuity improvement from baseline between groups. As a sensitivity analysis we used a generalized linear mixed model to allow for non-independence of visual acuity measurements over time and within patient but still did not find a statistically significant difference in visual acuity at 6 months between groups (P=0.11).

**Figure 2:**
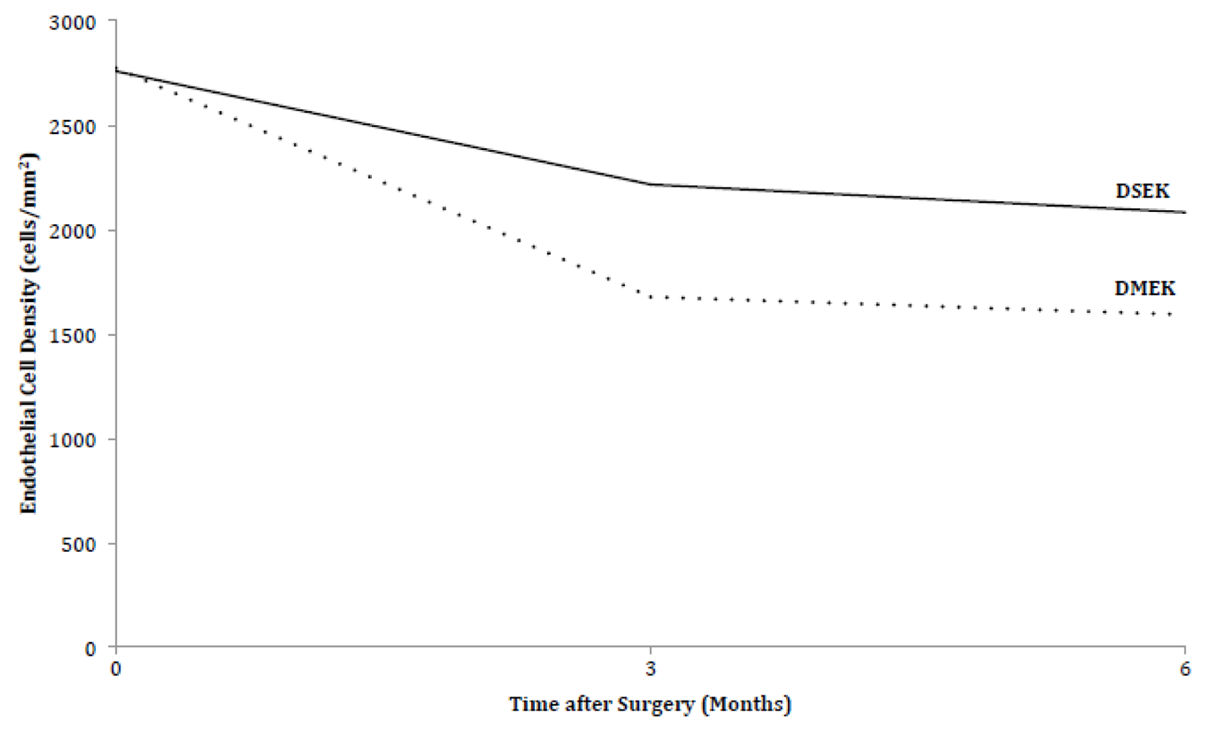
Endothelial Cell Loss. 6-month endothelial cell loss from baseline as measured by the Eye Bank in the study participants undergoing endothelial keratoplasty. DMEK: Descemet Membrane Endothelial Keratoplasty; DSEK: Descemet Stripping Endothelial Keratoplasty.

Even after excluding DMEK study participants with graft failure, DMEK patients had statistically significantly more endothelial cell loss. Mean 6-month cell counts were 2082 cells/mm^2^ (SD 492) in DSEK with a mean cell loss of 25%. In DMEK the mean 6-month cell counts were 1592 cells/mm^2^ (SD 547) with a mean cell loss of 43%. After excluding graft failures and controlling for baseline endothelial cell count as measured at the time of tissue preparation by the Eye Bank, DMEK patients lost on average 361 cells/mm^2^ more than DSEK at 3 months (95% CI −647 to −74; P=0.01) and 476 cells/mm^2^ more at 6 months (95% CI −758 to −194; P=0.001). Figure 2 compares 6-month endothelial cell loss from baseline between groups.

Complication rates between the two groups are compared in Table 3. There were 8 re-bubbles in the DMEK group and 1 in the DSEK group, however this difference was not statistically significant (P=0.12). There were 5 graft failures after DMEK compared with 0 after DSEK (P=0.09). There were no observed graft rejections in this study.

As a sensitivity analysis, to evaluate whether outcomes of DMEK improved over the duration of this study, we performed multiple linear regressions to analyze the association between date of DMEK surgery, as our primary predictor of interest with separate models for three different outcomes of interest including visual acuity, primary graft failure and post-operative endothelial cell counts. There was no difference in 6-month visual acuity (coef 0.00002, 95% CI −0.00015 to 0.0002, P=0.79) or 6-month endothelial cell counts (coef −0.098, 95% CI −0.51 to 0.30, P=0.63) over the duration of this study after controlling for baseline values. Similarly, the primary graft failure rate did not decrease over time (coef 0.0001, 95% CI −0.00007 to 0.0003), P=0.23)

## Discussion

In this study, we investigate outcomes of traditional DSEK versus DMEK prospectively in a single surgeon, consecutive case series during the surgeons DMEK learning curve. After controlling for baseline visual acuity, we found a statistically significant improvement in 3-month visual acuity in the DMEK group compared with the DSEK group of approximately one-half logMAR line, however, this difference lessened and was no longer statistically significant by six months. We also found a statistically significant increase in primary graft failure and decrease in 6-month endothelial cell counts, after controlling for baseline endothelial cell counts, in DMEK compared with DSEK. These data are important to inform both surgeons and their patients about outcomes during the surgeon’s DMEK learning curve given the number of corneal surgeons currently adopting this new technique.

A few large series in the literature report excellent outcomes with DMEK surgery after the surgeon-learning curve, which may be anywhere from 20 to 75 cases [6]. One such study by Melles’ group published results of a large cohort of 500 DMEK procedures performed by two surgeons and reported that 41% of study participants had visual acuity of 20/20 or better by 6 months, while 75% achieved a visual acuity of 20/25 or better [8]. In this series only 2.2% of patients required repeat grafting. A second Melles series, reported outcomes of 431 DMEKs during the surgeon learning curve performed by 18 experienced corneal surgeons in 11 countries [9]. In this study only 19% achieved visual acuity of 20/20 or better by 6 months, and only 43.8% of participants achieved visual acuity of 20/25 or better by 6 months. 156 (36%) of study participants were excluded from visual acuity analysis in this series because they had a second surgery, or had graft failure, which means that these data are likely biased toward favorable outcomes as they excluded a subset with poor initial surgical results. As might be expected during the learning curve, the re-bubble rate was much higher at 20% and 18% required re-operation, sometimes up to their 5th DMEK.

There were 5 (12%) primary graft failures in our series among the DMEK group, of these 4 went on to have repeat DSEK while 1 had repeat DMEK. Although there is some suggestion that outcomes after repeat endothelial keratoplasty are not as good,10 all of these patients did reasonably well with visual acuity between 20/20 and 20/40 at 3 and 6 months after their initial DMEK. In our analysis, we included the visual acuity of these patients despite the fact that they were re-grafted. Most of the literature on DMEK to date excludes graft failures from visual acuity analysis, which may lead to an over-optimistic picture of outcomes in DMEK. We felt that including the actual visual acuity results after re-graft gave a more fair representation of outcomes. Overall, in our study, visual acuity is excellent after both DSEK and DMEK, even during the surgeon’s DMEK learning curve. It is likely that the primary graft failure rate significantly decreases over time, although this was not observed in our study.

We also found that, after controlling for baseline endothelial cell counts and excluding graft failures, there was a statistically significant drop in endothelial cell counts after DMEK compared with DSEK [10]. Although some of this may be related to surgeon learning curve, endothelial cell loss did not improve over the course of our series. The corneal donor study found that 6-month endothelial cell counts of less than 1700 cells/mm^2^ were associated with a 41% 10-year graft failure rate in penetrating keratoplasty [11]. Therefore, the low 6-month endothelial cell counts observed in this study in the DMEK group are concerning. However, it is clear that patterns of cell loss are different in endothelial keratoplasty compared with penetrating keratoplasty with higher initial losses, followed by slower rates of decline after 6 months [12].

The strengths of this study include the fact that data were collected in a prospective fashion with consecutive patients without large baseline differences between DSEK and DMEK groups. It also demonstrates that good outcomes of DMEK can be achieved even during the surgeon learning curve. Limitations include the fact that the patients were not randomized and therefore there may have been some bias in patient selection, which could affect surgical outcomes. Also, comparing outcomes of one surgery during the learning curve to another that the surgeon is very experienced in may not be a fair comparison, although this experience is representative of the majority of EK surgeons in the United States at this time. DMEK standarized techniques evolved significantly during the time period of this study as did surgical technique of the surgeon (WC). As an example our own graft injection technique changed midway through the study. However, there was no difference in visual acuity outcomes, endothelial cell count, or complication rates between these two groups.

In summary, there may be faster visual recovery after DMEK surgery compared with DSEK, however, as expected during the surgeon learning curve, endothelial cell loss is worse and there are higher rates of complications such as primary graft failure. Given the recent EBAA statistics on tissue utilization for DMEK [4], it seems likely that many surgeons in the US either have not yet begun to perform the procedure or are on the early learning curve. If this study represents an average surgeon’s experience during the learning curve, then there may be a significant number of primary graft failures and successful grafts with low endothelial counts yet to be experienced in the US over the next several years. A randomized, controlled trial with surgeons comfortable and familiar with both techniques is necessary to determine which method is truly preferred.

## Figures and Tables

**Table 1:** Patient baseline demographics.

	DMEK N=42	DSEK N=22	P Value
Mean Age, years (SD)	69 (9.8)	68 (6.5)	0.55
Sex, Female (%)	20 (71)	7 (59)	0.53
Mean Baseline Visual Acuity, logMAR (SD)	0.38 (0.22)	0.35 (0.25)	0.42
Mean Baseline Pachymetry, μm (SD)	641 (63)	616 (40)	0.05
Mean Baseline Endothelial cell count, mm2 (SD)	2773 (224)	2757 (244)	0.82
Combined Surgery, # (%)^a^	21 (50)	17 (94)	<0.001*

Patient characteristics compared using Mann-Whitney U test due to differences in sample size between groups.

a Among DSEK additional procedures included iris-sutured IOL (1), phaco-emulsification with posterior chamber intraocular lens (16). Among DMEK additional procedures included phaco-emulsification with posterior chamber intraocular lens in all cases (21).

* Statistically significant difference after Holms-Šidák correction for multiple comparisons.

DMEK: Descemet Membrane Endothelial Keratoplasty; DSEK: Descemet Stripping Endothelial Keratoplasty; SD: Standard Deviation

**Table 2:** 6-Month visual acuity and endothelial cell density after endothelial keratoplasty

	DMEK Mean BSCVA (SD) (N=38)	DSEK Mean BSCVA (SD) (N=18)	Coefficient	95% CI	P Value
Visual Acuity, LogMAR^a^	0.09 (0.12)	0.14 (0.11)	−0.04	−0.10 to 0.02	0.22
Endothelial Cell Count^b^	1592 (547)	2082 (492)	−476	−758 to −194	0.001*

a Association between 6-month best spectacle-corrected visual acuity and type of endothelial keratoplasty analyzed using multiple linear regression, controlling for baseline best spectacle-corrected visual acuity with intent to treat analysis including 3 and 6 month visual acuity after initial endothelial keratoplasty even if re-grafted.

b Association between 6-month endothelial cell count and type of endothelial keratoplasty analyzed using multiple linear regressions, controlling for baseline endothelial cell count as measured by the eyebank. Note that 5 grafts in DMEK arm were excluded from analysis due to graft failure.

* Statistically significant difference after Holms-Šidák correction for multiple comparisons.

DMEK: Descemet Membrane Endothelial Keratoplasty; DSEK: Descemet Stripping Endothelial Keratoplasty; BSCVA: Best Spectacle-Corrected Visual Acuity; SD: Standard Deviation; 95% CI, 95% Confidence Intervals

**Table 3:** Complication Rates in DMEK compared with DSEK.

	DMEK Number (%)	DSEK Number (%)	P Value
Re-bubble	8 (19)	1 (5.6)	0.12
Graft Failure	5 (12)	0 (0)	0.09
Graft Rejection	0 (0)	0 (0)	1.0
Glaucoma	1 (2.4)	0 (0)	0.70
CMEa	2 (4.7)	0 (0)	0.48

Complication rates compared using T-test adjusting for unequal variances due to differences in sample size between groups.

DMEK: Descemet Membrane Endothelial Keratoplasty; DSEK: Descemet Stripping Endothelial Keratoplasty; CME: Cystoid Macular Edema
